# Comparative outcomes of adjuvant treatment approaches in resected stage I-III gastric cancer

**DOI:** 10.3389/fonc.2026.1933478

**Published:** 2026-10-20

**Authors:** Bulent Yildiz, Yasemin Sagdic Karateke, Duygu Bayir Garbioglu, Metin Demir

**Affiliations:** Department of Medical Oncology, Faculty of Medicine, Eskisehir Osmangazi University, Eskisehir, Türkiye

**Keywords:** adjuvant treatment, cisplatin-based chemotherapy, disease-free survival, gastric cancer, overall survival, XELOX

## Abstract

**Background:**

Gastric cancer remains a major cause of cancer-related mortality. We evaluated clinicopathological prognostic factors and compared commonly used adjuvant treatment approaches after curative resection.

**Methods:**

This retrospective study included 277 patients with stage I-III gastric cancer treated with adjuvant chemotherapy with or without chemoradiotherapy between 2006 and 2022. Treatment groups were defined by systemic chemotherapy regimen: fluorouracil plus folinic acid (FUFA), capecitabine plus oxaliplatin (XELOX), or cisplatin-based chemotherapy. Concurrent chemoradiotherapy was administered to 98.7%, 81.0%, and 92.9% of patients, respectively.

**Results:**

The FUFA, XELOX, and cisplatin-based groups comprised 156, 58, and 28 patients. Advanced stage, ECOG performance status ≥2, R1 resection, extracapsular extension, high grade, lymphatic invasion, perineural invasion, tumor nodules, and treatment discontinuation were associated with shorter disease-free survival (DFS) and overall survival (OS). In multivariable analysis, pathological stage and perineural invasion remained independently associated with survival. Compared with FUFA, XELOX was associated with lower hazards of recurrence (HR = 0.48, 95% CI: 0.29–0.80; p=0.005) and mortality (HR = 0.48, 95% CI: 0.28–0.82; p=0.007), whereas the cisplatin-based approach did not differ significantly from the FUFA reference group. When treatment period was forced into the models, the DFS association for XELOX was no longer statistically significant (HR = 0.40, 95% CI: 0.15–1.04; p=0.060), whereas the OS association remained significant (HR = 0.32, 95% CI: 0.12–0.87; p=0.026). In unadjusted pairwise comparisons, FUFA and XELOX did not differ significantly in DFS or OS in the overall cohort. In the exploratory analysis of node-positive patients, XELOX and FUFA also did not differ significantly in DFS or OS (p=0.088 and p=0.132). All-grade neutropenia, diarrhea, and neuropathy differed significantly among treatment groups; no treatment-related deaths occurred.

**Conclusions:**

FUFA and XELOX did not differ significantly in DFS or OS in unadjusted pairwise comparisons overall or in the exploratory analysis of node-positive patients. However, XELOX was associated with lower recurrence and mortality hazards in the primary multivariable models; in the treatment-period-forced sensitivity analysis, the DFS association was no longer statistically significant, whereas the OS association remained significant. This discordance for DFS should be interpreted cautiously because treatment approach and treatment period were closely linked, making a clear causal interpretation difficult.

## Introduction

Gastric cancer remains an important cause of cancer-related morbidity and mortality worldwide despite a gradual decline in its incidence over recent decades. According to GLOBOCAN 2022 estimates, gastric cancer is the fifth most frequently diagnosed malignancy and the fifth leading cause of cancer-related death worldwide (1). Although long-term survival is favorable in early-stage disease, outcomes deteriorate substantially with increasing stage, particularly in stage II and III disease (2).

Several clinicopathological factors have been associated with recurrence and survival after curative resection, including pathological stage (2), lymph node involvement (3), extent of lymphadenectomy (4, 5), tumor grade (6), and the presence of lymphovascular or perineural invasion (7). Because of the considerable risk of recurrence following surgery alone, multimodality treatment strategies have been developed to improve long-term outcomes.

Current guidelines recommend adjuvant treatment for patients with node-positive disease or locally advanced tumors after curative resection. Depending on the extent of lymph node dissection and regional treatment practices, postoperative treatment may consist of chemotherapy alone or chemoradiotherapy. Capecitabine plus oxaliplatin (XELOX), fluoropyrimidine-based regimens, and S-1-based approaches are among the commonly used adjuvant treatment options (8–11). However, the optimal postoperative treatment approach has not been clearly established, particularly in real-world clinical practice.

Several randomized studies have demonstrated the benefit of individual adjuvant strategies compared with surgery alone or have compared selected regimens with each other (8–11). However, data directly comparing commonly used adjuvant treatment approaches in routine clinical practice remain limited.

In the present study, we evaluated the association between clinicopathological characteristics and survival outcomes in patients with resected stage I-III gastric cancer and compared the efficacy and toxicity profiles of the three most frequently used adjuvant treatment approaches, defined according to the systemic chemotherapy regimen administered, in our institution: fluorouracil plus folinic acid (FUFA), capecitabine plus oxaliplatin (XELOX), and cisplatin-based chemotherapy.

## Materials and methods

### Study population

This retrospective cohort study included 277 patients with stage I-III gastric adenocarcinoma who underwent subtotal or total gastrectomy followed by adjuvant chemotherapy with or without chemoradiotherapy at the Department of Medical Oncology, Eskisehir Osmangazi University, between January 2006 and December 2022.

To account for temporal changes in adjuvant treatment practice over the long study period, treatment period was categorized by year of surgery as 2006–2016 and 2017–2022. Demographic characteristics, laboratory findings, clinicopathological features, treatment information, and treatment-related toxicities were retrospectively collected from medical records. Associations between these variables and disease-free survival (DFS) and overall survival (OS) were evaluated. Survival outcomes and toxicity profiles were also compared according to the administered adjuvant treatment approach, defined by the systemic chemotherapy regimen administered.

Patients who did not undergo curative surgery, received neoadjuvant treatment, had non-adenocarcinoma histology, or had metastatic disease at diagnosis were excluded. Tumors were staged according to the 8th edition of the American Joint Committee on Cancer (AJCC) TNM staging system. Patients were identified consecutively from institutional medical records. Patients had undergone upfront surgery either at our institution or at outside centers and were subsequently referred to medical oncology for adjuvant treatment. Because preoperative staging information was not consistently available in the retrospective records, clinical cT and cN categories could not be reliably assessed.

### Treatment

Three adjuvant treatment groups, defined according to the systemic chemotherapy regimen administered, were evaluated: fluorouracil plus folinic acid (FUFA), capecitabine plus oxaliplatin (XELOX), and cisplatin-based chemotherapy.

The FUFA regimen consisted of fluorouracil 425 mg/m² plus folinic acid 20 mg/m² administered daily for 5 consecutive days, followed by concurrent chemoradiotherapy with fluorouracil 400 mg/m² and folinic acid 20 mg/m² during the first four and last three days of radiotherapy. After completion of radiotherapy, two additional 5-day cycles of FUFA were administered. Concurrent chemoradiotherapy was administered to 154 patients (98.7%) in this group.

The XELOX regimen consisted of oxaliplatin 130 mg/m² on day 1 and capecitabine 1000 mg/m² twice daily on days 1–14 every 3 weeks. Patients received between three and eight cycles of treatment. Concurrent chemoradiotherapy was administered to 47 patients (81.0%) in this group.

The cisplatin-based group received either cisplatin 20 mg/m² and fluorouracil 1000 mg/m² on days 1–4 every 4 weeks for up to eight cycles or cisplatin 75 mg/m² on day 1 plus capecitabine 1000 mg/m² twice daily on days 1–14 every 4 weeks. Patients received three to six cycles of treatment, and 26 of 28 patients (92.9%) underwent concurrent chemoradiotherapy. Treatment completion and discontinuation status was retrospectively assessed from medical records; patients with missing treatment-completion data were excluded from the corresponding between-group comparison. Because of the retrospective nature of the study, the specific clinical rationale for selecting the systemic chemotherapy regimen or administering chemoradiotherapy to individual patients could not be reliably reconstructed from the medical records. Treatment choices reflected routine clinical practice during the study period and changed over time.

### Statistical analysis

Statistical analyses were performed using IBM SPSS Statistics for Windows, version 25.0 (IBM Corp., Armonk, NY, USA). Disease-free survival was defined as the time from surgery to disease recurrence or death from any cause, whichever occurred first. Overall survival was defined as the time from surgery to death from any cause or last follow-up.

Survival curves were estimated using the Kaplan–Meier method and compared using the log-rank test. When the overall log-rank test showed a significant difference among adjuvant treatment groups, pairwise log-rank comparisons were performed. An exploratory subgroup analysis was also performed in patients with lymph node-positive disease. Associations between categorical variables were evaluated using Pearson’s chi-square test. For toxicity comparisons with sparse cell counts, the Fisher–Freeman–Halton exact test with Monte Carlo simulation was used.

Univariable analyses were performed using available-case data, with patients who had missing values excluded from the corresponding analysis. Baseline variables with a p value <0.05 in univariable analyses were considered for inclusion in multivariable Cox proportional hazards regression models using the backward stepwise method. ECOG performance status and surgical margin status were included among the candidate covariates in the multivariable models. Treatment completion was not included in the Cox models because it was a post-baseline variable; treatment discontinuation could occur because of disease progression or death before completion of treatment. Multivariable analyses were performed in patients with complete data for the candidate covariates. Because adjuvant treatment approach was strongly associated with treatment period, an additional sensitivity analysis was performed in which treatment period (2006–2016 vs 2017–2022) was forced into the final multivariable Cox models irrespective of its univariable significance. An exploratory, unadjusted Cox analysis also compared XELOX with FUFA among patients treated during 2017–2022.

The proportional hazards assumption for the Cox regression models was assessed using time-dependent covariates based on interactions between the covariates and the natural logarithm of survival time. A two-sided p value <0.05 was considered statistically significant.

### Ethics approval

This study was approved by the Ethics Committee of Eskisehir Osmangazi University Faculty of Medicine (Approval No. 34/2024; March 19, 2024). Informed consent was waived because of the retrospective study design, and the study was conducted in accordance with the Declaration of Helsinki.

## Results

### Patient characteristics

A total of 277 patients with stage I-III gastric cancer who underwent curative surgery followed by adjuvant treatment were included in the study. The median follow-up duration was 33.1 months (range, 3.0–223.0 months). The median age at diagnosis was 61 years (range, 22–81 years), and the median tumor size was 5.2 cm (range, 1–25 cm). The median disease-free survival (DFS) and overall survival (OS) for the entire cohort were 24.9 months (95% CI, 18.8–30.9 months) and 39.0 months (95% CI, 32.4–45.6 months), respectively.

Among all patients, 156 (56.3%) received FUFA, 58 (20.9%) received XELOX, and 28 (10.1%) received cisplatin-based chemotherapy. The median follow-up durations in the FUFA, XELOX, cisplatin-based, and other-regimen groups were 40.1, 35.3, 24.3, and 29.0 months, respectively.

The associations between clinicopathological characteristics and survival outcomes are summarized in **Table 1**. ECOG performance status ≥2 was associated with shorter DFS and OS compared with ECOG 0–1 (p=0.007 and p=0.006, respectively). R1 resection was also associated with shorter DFS and OS than R0 resection (both p<0.001). Treatment discontinuation was associated with shorter DFS and OS in univariable analyses (both p<0.001). Treatment period was not significantly associated with DFS or OS (p=0.258 and p=0.393, respectively). Subtotal gastrectomy was associated with significantly longer DFS and OS than total gastrectomy (p=0.016 and p=0.006, respectively). Increasing pathological stage was associated with progressively shorter survival, and patients with stage IIIC disease had the poorest outcomes (both p<0.001). The presence of lymph node involvement and extracapsular extension was also associated with shorter DFS and OS (both p<0.001). In addition, high tumor grade, vascular invasion, lymphatic invasion, perineural invasion, and tumor nodules were significantly associated with worse DFS and OS.

**Table 1:** Associations between clinicopathological characteristics and survival outcomes in the study cohort.

Variable	n (%)	Median DFS, months (95% CI)	p value	Median OS, months (95% CI)	p value
Gender			0.321		0.384
Male	199 (71.8)	23.9 (17.8–30.0)		37.0 (27.7–46.3)	
Female	78 (28.2)	31.4 (14.9–47.8)		41.6 (27.7–55.5)	
Age			0.258		0.371
≤50 years	50 (18.1)	24.8 (15.9–33.7)		33.2 (25.1–41.4)	
>50 years	227 (81.9)	24.9 (17.7–32.1)		39.7 (31.4–47.9)	
ECOG performance status			0.007		0.006
0–1	179 (64.6)	35.7 (20.0–51.5)		51.2 (39.2–63.2)	
≥2	39 (14.1)	17.2 (6.0–28.5)		29.9 (14.3–45.5)	
Missing	59 (21.3)	—		—	
Treatment period			0.258		0.393
2006–2016	186 (67.1)	22.3 (16.0–28.5)		33.1 (24.9–41.3)	
2017–2022	91 (32.9)	33.5 (17.4–49.6)		45.6 (37.4–53.8)	
Tumor localization			0.223		0.261
Upper third	61 (22.0)	20.4 (15.3–25.5)		28.7 (19.3–38.0)	
Middle third	167 (60.3)	29.9 (18.0–41.8)		41.6 (28.3–55.0)	
Lower third	49 (17.7)	33.1 (14.9–51.2)		40.5 (24.1–57.0)	
Operation type			0.016		0.006
Subtotal gastrectomy	114 (41.2)	35.6 (6.1–65.2)		45.8 (27.1–64.4)	
Total gastrectomy	163 (58.8)	21.7 (16.6–26.9)		31.8 (24.1–39.5)	
Surgical margin status			<0.001		<0.001
R0	231 (83.4)	32.0 (22.0–42.1)		43.0 (33.4–52.5)	
R1	46 (16.6)	15.1 (13.7–16.6)		21.1 (13.8–28.5)	
Pathological stage			<0.001		<0.001
I	19 (6.9)	139.2 (43.7–234.8)		139.2 (113.7–164.8)	
II	64 (23.1)	136.7 (90.5–183.0)		136.7 (106.5–167.0)	
IIIA	88 (31.8)	24.4 (17.5–31.3)		39.0 (29.8–48.2)	
IIIB	61 (22.0)	18.8 (12.7–24.9)		30.9 (19.8–41.9)	
IIIC	45 (16.2)	11.0 (9.1–12.9)		19.4 (16.8–21.9)	
Lymph node involvement			<0.001		<0.001
Absent	44 (15.9)	145.7 (78.2–213.2)		132.2 (83.4–181.0)	
Present	233 (84.1)	20.6 (16.1–25.2)		32.9 (27.4–38.5)	
Number of dissected lymph nodes			0.489		0.968
≤15	85 (30.7)	21.3 (14.4–28.3)		39.0 (24.0–54.0)	
>15	192 (69.3)	28.0 (18.7–37.2)		37.9 (31.0–44.9)	
Extracapsular involvement			<0.001		<0.001
Negative	167 (60.3)	50.3 (18.0–82.6)		66.9 (33.0–100.7)	
Positive	110 (39.7)	14.9 (12.7–17.0)		24.8 (19.6–30.1)	
Tumor grade			0.018		0.007
Grade 1–2	129 (46.6)	30.2 (11.4–49.0)		52.4 (32.3–72.4)	
Grade 3	148 (53.4)	19.8 (13.8–25.8)		28.3 (21.1–35.4)	
Vascular invasion			0.031		0.006
Negative	116 (41.9)	28.7 (14.6–42.8)		43.0 (28.0–58.0)	
Positive	156 (56.3)	22.4 (15.6–29.3)		32.0 (24.8–39.2)	
Missing	5 (1.8)	—		—	
Perineural invasion			<0.001		<0.001
Negative	76 (27.4)	66.9 (0.2–133.5)		81.5 (12.1–150.9)	
Positive	195 (70.4)	21.3 (16.3–26.3)		29.9 (23.8–36.0)	
Missing	6 (2.2)	—		—	
Lymphatic invasion			0.001		<0.001
Negative	64 (23.1)	78.3 (0.0–165.4)		101.6 (48.0–155.2)	
Positive	207 (74.7)	21.7 (16.4–27.0)		32.0 (25.8–38.3)	
Missing	6 (2.2)	—		—	
Tumor nodule			0.013		0.008
Absent	219 (79.1)	29.9 (19.4–40.3)		39.8 (28.0–51.6)	
Present	49 (17.7)	17.6 (14.1–21.1)		28.3 (18.4–38.1)	
Missing	9 (3.2)	—		—	
Treatment completion			<0.001		<0.001
Completed	218 (78.7)	30.0 (20.6–39.4)		43.0 (32.4–53.6)	
Discontinued	33 (11.9)	7.9 (4.6–11.2)		12.9 (5.4–20.4)	
Missing	26 (9.4)	—		—	
Adjuvant treatment approach			0.010		0.022
FUFA	156 (56.3)	27.4 (14.0–38.4)		40.5 (26.0–53.4)	
XELOX	58 (20.9)	39.0 (22.4–55.6)		45.8 (36.7–54.9)	
Cisplatin-based	28 (10.1)	17.5 (10.7–21.0)		24.4 (18.4–30.0)	
Other regimens	35 (12.6)	—		—	

CI, confidence interval; DFS, disease-free survival; OS, overall survival; FUFA, fluorouracil plus folinic acid; XELOX, capecitabine plus oxaliplatin. Survival distributions were estimated using the Kaplan–Meier method and compared using the log-rank test. Percentages were calculated using the total study population (N = 277) as the denominator. Missing data are shown separately and were excluded from survival comparisons. Survival comparisons according to adjuvant treatment approach were restricted to FUFA, XELOX, and cisplatin-based chemotherapy (n=242).

### Survival outcomes

Survival differed among the three treatment groups in the overall log-rank comparisons (p=0.010 for DFS and p=0.022 for OS), with the shortest median survival observed in the cisplatin-based group. Pairwise comparisons showed no significant differences between FUFA and XELOX (p=0.592 for DFS and p=0.734 for OS) (**Figure 1**).

**Figure 1:**
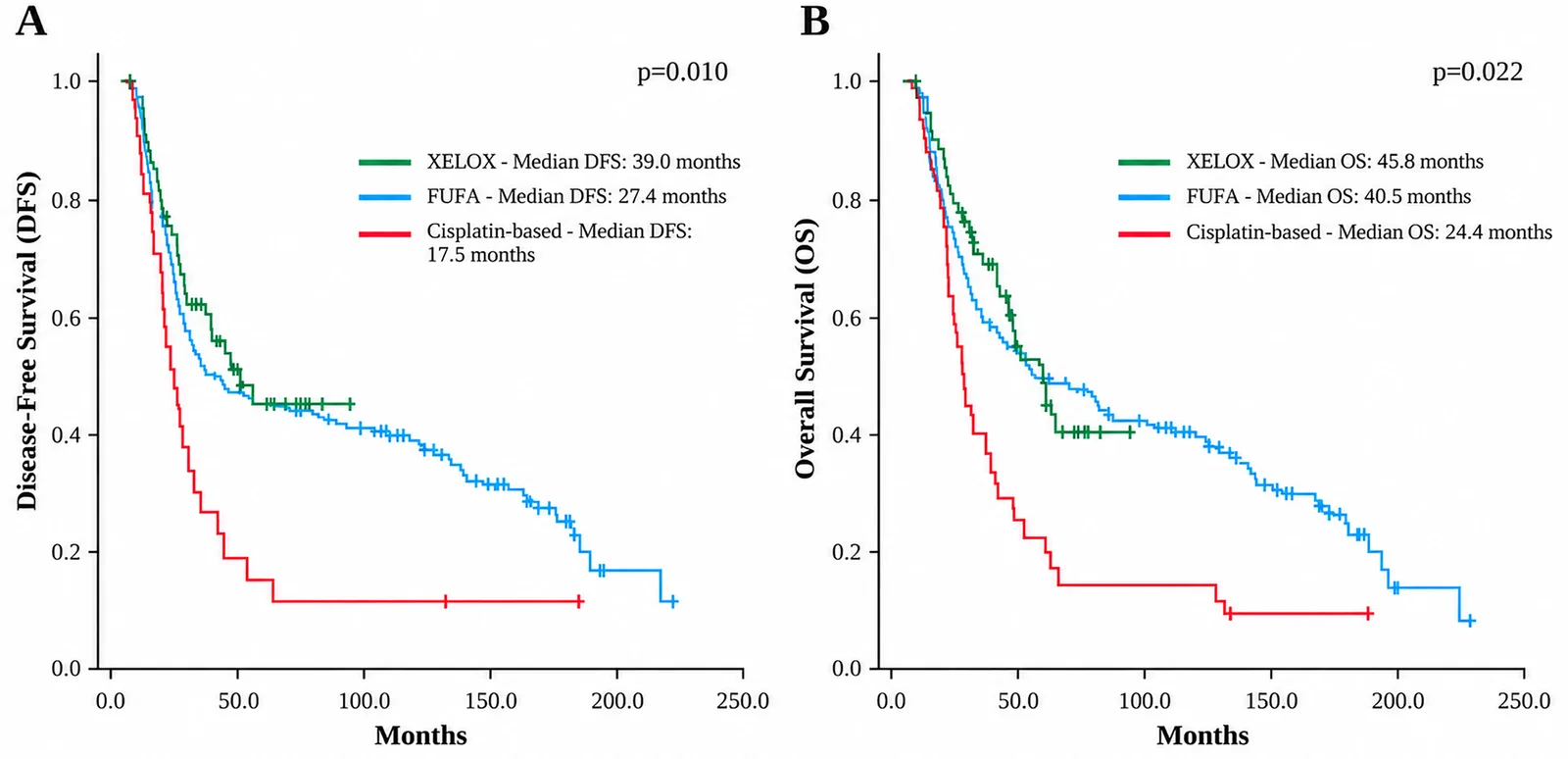
Unadjusted Kaplan-Meier curves for disease-free survival **(A)** and overall survival **(B)** according to adjuvant treatment group, defined by systemic chemotherapy regimen (FUFA, XELOX, and cisplatin-based chemotherapy) in patients with resected stage I-III gastric cancer. The displayed p values are from overall log-rank comparisons among the three groups.

Baseline clinicopathological variables that were significant in univariable analyses were entered into multivariable Cox regression models using a backward stepwise procedure. The multivariable analyses included 177 patients with complete data for the candidate covariates. ECOG performance status, surgical margin status, operation type, extracapsular involvement, tumor grade, vascular invasion, and lymphatic invasion were not retained in the final models. Pathological stage remained independently associated with both DFS and OS (global p<0.001 for both). Compared with stage I, stage IIIC disease was associated with higher hazards of recurrence (HR = 8.26, 95% CI: 3.20–21.30; p<0.001) and mortality (HR = 7.44, 95% CI: 2.83–19.55; p<0.001). Stage IIIB was not significantly associated with DFS or OS after adjustment (HR = 2.07, 95% CI: 0.83–5.14; p=0.117 and HR = 2.21, 95% CI: 0.89–5.49; p=0.088, respectively). Perineural invasion was also independently associated with poorer DFS and OS (HR = 1.93, 95% CI: 1.21–3.09; p=0.006 and HR = 2.03, 95% CI: 1.27–3.26; p=0.003, respectively). Adjuvant treatment approach remained significant in both models (global p=0.009 for DFS and p=0.011 for OS). Compared with FUFA, XELOX was associated with lower hazards of recurrence (HR = 0.48, 95% CI: 0.29–0.80; p=0.005) and mortality (HR = 0.48, 95% CI: 0.28–0.82; p=0.007), whereas no significant difference was observed between the cisplatin-based approach and FUFA. Tumor nodule status was retained only in the OS model but did not reach statistical significance (HR = 1.52, 95% CI: 0.97–2.39; p=0.068) (**Table 2**). No significant violations of the proportional hazards assumption were identified for the final DFS or OS models.

**Table 2:** Multivariable Cox regression analysis of factors associated with disease-free and overall survival.

Variables	DFS HR (95% CI)	p value	OS HR (95% CI)	p value
Pathological stage		<0.001*		<0.001*
I	Reference	—	Reference	—
II	0.91 (0.37–2.26)	0.837	0.95 (0.38–2.38)	0.921
IIIA	2.00 (0.82–4.86)	0.126	1.91 (0.78–4.65)	0.155
IIIB	2.07 (0.83–5.14)	0.117	2.21 (0.89–5.49)	0.088
IIIC	8.26 (3.20–21.30)	<0.001	7.44 (2.83–19.55)	<0.001
Perineural invasion				
Negative	Reference	—	Reference	—
Positive	1.93 (1.21–3.09)	0.006	2.03 (1.27–3.26)	0.003
Tumor nodule				
Absent	—	—	Reference	—
Present	—	—	1.52 (0.97–2.39)	0.068
Adjuvant treatment approach		0.009*		0.011*
FUFA	Reference	—	Reference	—
XELOX	0.48 (0.29–0.80)	0.005	0.48 (0.28–0.82)	0.007
Cisplatin-based	1.06 (0.64–1.75)	0.826	1.09 (0.66–1.79)	0.741

CI, confidence interval; HR, hazard ratio; DFS, disease-free survival; OS, overall survival; FUFA, fluorouracil plus folinic acid; XELOX, capecitabine plus oxaliplatin. * Global p values are reported for pathological stage and adjuvant treatment approach. The table presents variables retained in the final backward stepwise Cox regression models.

Because adjuvant treatment approach was strongly associated with treatment period, an additional sensitivity analysis was performed by forcing treatment period (2006–2016 vs 2017–2022) into the final multivariable Cox models. In this analysis, treatment period was not independently associated with DFS (HR = 1.23, 95% CI: 0.51–2.94; p=0.649) or OS (HR = 1.51, 95% CI: 0.62–3.67; p=0.360). The point estimate for XELOX compared with FUFA remained below unity for both DFS (HR = 0.40, 95% CI: 0.15–1.04; p=0.060) and OS (HR = 0.32, 95% CI: 0.12–0.87; p=0.026). However, treatment approach and treatment period were highly correlated, with 57 of 58 XELOX-treated patients receiving treatment during 2017–2022, limiting the ability of the model to separate treatment effects from temporal changes in clinical practice.

In an exploratory comparison restricted to 2017–2022, 57 patients received XELOX and 11 received FUFA. The unadjusted XELOX versus FUFA estimates were HR = 0.97 (95% CI: 0.43–2.21; p=0.947) for DFS and HR = 1.01 (95% CI: 0.44–2.30; p=0.985) for OS.

### Subgroup analysis

The distribution of clinicopathological characteristics according to treatment groups is shown in **Table 3**. Significant differences were observed among the treatment groups with respect to ECOG performance status, treatment period, tumor location, pathological N stage, pathological stage, number of dissected lymph nodes, extracapsular involvement, vascular invasion, lymphatic invasion, tumor nodules, surgical margin status, and receipt of chemoradiotherapy. ECOG performance status was available for 218 of 277 patients (78.7%); among patients with available ECOG data, ECOG ≥2 was more frequent in the cisplatin-based group (p=0.030). Adjuvant treatment approach was strongly associated with treatment period (p<0.001); 57 of 58 patients (98.3%) receiving XELOX were treated during 2017–2022, whereas all 28 patients receiving cisplatin-based chemotherapy were treated during 2006–2016. R1 resection was more frequent in the cisplatin-based group (35.7%) than in the FUFA (12.2%) and XELOX (10.3%) groups (p=0.003). Operation type did not differ significantly among the treatment groups (p=0.568), whereas the number of dissected lymph nodes differed significantly (p<0.001). Among patients with available treatment-completion data, completion rates were 87.4% (132/151) in the FUFA group, 80.9% (38/47) in the XELOX group, and 88.5% (23/26) in the cisplatin-based group, with no significant difference among the groups (p=0.490). Because of these baseline differences, an exploratory subgroup analysis was performed in 233 patients with lymph node-positive disease. Among the 202 node-positive patients who received FUFA, XELOX, or cisplatin-based chemotherapy, there was no significant difference in stage distribution among the three treatment groups (p=0.184). Kaplan–Meier analysis showed significant differences among the three treatment groups in both DFS and OS (log-rank p=0.025 and p=0.031, respectively). Pairwise comparisons showed no significant differences between XELOX and FUFA in either DFS or OS (p=0.088 and p=0.132, respectively), whereas patients receiving cisplatin-based chemotherapy had shorter DFS and OS than those receiving XELOX (p=0.003 and p=0.004, respectively).

**Table 3:** Baseline clinicopathological characteristics according to adjuvant treatment group.

Characteristic	FUFA (n=156), n (%)	XELOX (n=58), n (%)	Cisplatin-based (n=28), n (%)	p value
Gender				0.912
Male	112 (71.8)	41 (70.7)	19 (67.9)	
Female	44 (28.2)	17 (29.3)	9 (32.1)	
Age				0.700
≤50 years	32 (20.5)	9 (15.5)	5 (17.9)	
>50 years	124 (79.5)	49 (84.5)	23 (82.1)	
ECOG performance status				0.030
0-1	95 (60.9)	44 (75.9)	17 (60.7)	
≥2	14 (9.0)	9 (15.5)	9 (32.1)	
Missing	47 (30.1)	5 (8.6)	2 (7.1)	
Treatment period				<0.001
2006–2016	145 (92.9)	1 (1.7)	28 (100.0)	
2017–2022	11 (7.1)	57 (98.3)	0 (0.0)	
Tumor localization				<0.001
Upper third	32 (20.5)	15 (25.9)	9 (32.1)	
Middle third	112 (71.8)	18 (31.0)	18 (64.3)	
Lower third	12 (7.7)	25 (43.1)	1 (3.6)	
Operation type				0.568
Subtotal gastrectomy	65 (41.7)	22 (37.9)	14 (50.0)	
Total gastrectomy	91 (58.3)	36 (62.1)	14 (50.0)	
Surgical margin status				0.003
R0	137 (87.8)	52 (89.7)	18 (64.3)	
R1	19 (12.2)	6 (10.3)	10 (35.7)	
Pathological T stage				0.103
pT1	11 (7.1)	2 (3.4)	1 (3.6)	
pT2	13 (8.3)	2 (3.4)	1 (3.6)	
pT3	76 (48.7)	31 (53.4)	8 (28.6)	
pT4	56 (35.9)	23 (39.7)	18 (64.3)	
Pathological N stage				0.005
pN0	35 (22.4)	2 (3.4)	3 (10.7)	
pN1	32 (20.5)	9 (15.5)	3 (10.7)	
pN2	44 (28.2)	21 (36.2)	6 (21.4)	
pN3a	33 (21.2)	15 (25.9)	10 (35.7)	
pN3b	12 (7.7)	11 (19.0)	6 (21.4)	
Pathological stage				0.007
I	14 (9.0)	0 (0.0)	1 (3.6)	
II	48 (30.8)	10 (17.2)	2 (7.1)	
IIIA	49 (31.4)	20 (34.5)	10 (35.7)	
IIIB	30 (19.2)	16 (27.6)	9 (32.1)	
IIIC	15 (9.6)	12 (20.7)	6 (21.4)	
Number of dissected lymph nodes				<0.001
≤15	62 (39.7)	6 (10.3)	11 (39.3)	
>15	94 (60.3)	52 (89.7)	17 (60.7)	
Extracapsular involvement				<0.001
Negative	95 (60.9)	45 (77.6)	9 (32.1)	
Positive	61 (39.1)	13 (22.4)	19 (67.9)	
Tumor grade				0.175
Grade 1–2	83 (53.2)	26 (44.8)	10 (35.7)	
Grade 3	73 (46.8)	32 (55.2)	18 (64.3)	
Vascular invasion				<0.001
Negative	93 (59.6)	7 (12.1)	16 (57.1)	
Positive	60 (38.5)	50 (86.2)	12 (42.9)	
Missing	3 (1.9)	1 (1.7)	0 (0.0)	
Perineural invasion				0.151
Negative	52 (33.3)	12 (20.7)	6 (21.4)	
Positive	102 (65.4)	43 (74.1)	22 (78.6)	
Missing	2 (1.3)	3 (5.2)	0 (0.0)	
Lymphatic invasion				0.009
Negative	51 (32.7)	8 (13.8)	5 (17.9)	
Positive	101 (64.7)	49 (84.5)	23 (82.1)	
Missing	4 (2.6)	1 (1.7)	0 (0.0)	
Tumor nodule				0.044
Absent	133 (85.3)	41 (70.7)	21 (75.0)	
Present	19 (12.2)	14 (24.1)	7 (25.0)	
Missing	4 (2.6)	3 (5.2)	0 (0.0)	
Treatment completion				0.490
Completed	132 (84.6)	38 (65.5)	23 (82.1)	
Discontinued	19 (12.2)	9 (15.5)	3 (10.7)	
Missing	5 (3.2)	11 (19.0)	2 (7.1)	
Chemoradiotherapy				<0.001
No	2 (1.3)	11 (19.0)	2 (7.1)	
Yes	154 (98.7)	47 (81.0)	26 (92.9)	

Values are presented as n (%). Percentages were calculated using the total number of patients in each treatment group as the denominator. Missing data are shown separately and were excluded from the chi-square tests. FUFA, fluorouracil plus folinic acid; XELOX, capecitabine plus oxaliplatin. The p value for ECOG performance status was calculated among patients with available ECOG data; patients with missing ECOG data were excluded from the comparison. Treatment completion was assessed among patients with available treatment-discontinuation data; missing data are shown separately and were excluded from the between-group comparison. Discontinuation included treatment cessation for clinical reasons, disease progression, or death before completion. R0 was defined as a microscopically negative resection margin, including both margins ≥1 cm and close (<1 cm) but microscopically negative margins; R1 was defined as a microscopically positive resection margin. No R2 resections were recorded in the study cohort.

### Treatment-related toxicity

Treatment-related toxicities are summarized in **Table 4**. Because of incomplete toxicity documentation in the retrospective medical records, the number of evaluable patients varied across adverse events, ranging from 70 to 107 in the FUFA group, 27 to 45 in the XELOX group, and 12 to 17 in the cisplatin-based group. Rates of all-grade neutropenia, diarrhea, and neuropathy differed significantly among the treatment groups (p=0.028, p=0.045, and p=0.003, respectively). Neutropenia and diarrhea were numerically most frequent in the cisplatin-based group, whereas neuropathy was most frequent in the XELOX group. No significant differences were observed among the treatment groups in the rates of grade 3–4 hematological or non-hematological toxicities. No treatment-related deaths occurred in any treatment group.

**Table 4:** Treatment-related toxicities according to adjuvant treatment group.

Adverse event	All-grade adverse events				Grade 3–4 adverse events			
	FUFA n/N (%)	XELOX n/N (%)	Cisplatin-based n/N (%)	P value	FUFA n/N (%)	XELOX n/N (%)	Cisplatin-based n/N (%)	P value
Hematologic								
Febrile neutropenia	3/107 (2.8)	1/45 (2.2)	1/17 (5.9)	0.606	3/107 (2.8)	1/45 (2.2)	1/17 (5.9)	0.606
Anemia	62/107 (57.9)	24/45 (53.3)	11/17 (64.7)	0.706	0/107 (0)	0/45 (0)	1/17 (5.9)	0.101
Leukopenia	45/107 (42.1)	24/45 (53.3)	12/17 (70.6)	0.060	5/107 (4.7)	2/45 (4.4)	3/17 (17.6)	0.136
Neutropenia	24/107 (22.4)	14/45 (31.1)	9/17 (52.9)	0.028	4/107 (3.7)	1/45 (2.2)	2/17 (11.8)	0.312
Thrombocytopenia	32/107 (29.9)	21/45 (46.7)	8/17 (47.1)	0.089	4/107 (3.7)	3/45 (6.7)	1/17 (5.9)	0.544
Non-hematologic								
Neuropathy	2/90 (2.2)	8/41 (19.5)	1/15 (6.7)	0.003	0/90 (0)	1/41 (2.4)	0/15 (0)	0.384
Diarrhea	3/83 (3.6)	2/39 (5.1)	3/14 (21.4)	0.045	0/83 (0)	1/39 (2.6)	0/14 (0)	0.390
ALT elevation	24/106 (22.6)	12/44 (27.3)	3/17 (17.6)	0.701	3/106 (2.8)	0/44 (0)	0/17 (0)	0.679
AST elevation	23/106 (21.7)	12/44 (27.3)	1/17 (5.9)	0.194	4/106 (3.8)	0/44 (0)	0/17 (0)	0.560
Total bilirubin elevation	14/107 (13.1)	6/44 (13.6)	1/17 (5.9)	0.682	2/107 (1.9)	0/44 (0)	0/17 (0)	1.000
Nausea/vomiting	34/70 (48.6)	16/27 (59.3)	8/12 (66.7)	0.400	4/70 (5.7)	2/27 (7.4)	2/12 (16.7)	0.318
Hand-foot syndrome	2/106 (1.9)	0/39 (0)	0/17 (0)	1.000	1/106 (0.9)	0/39 (0)	0/17 (0)	1.000
Creatinine elevation	5/105 (4.8)	0/44 (0)	1/17 (5.9)	0.332	0/105 (0)	0/44 (0)	0/17 (0)	–

ALT, alanine aminotransferase; AST, aspartate aminotransferase; FUFA, fluorouracil plus folinic acid; XELOX, capecitabine plus oxaliplatin. Data are presented as n/N (%), where N represents the number of patients with available data for the respective adverse event. Patients with missing toxicity data were excluded from the corresponding analysis. Because toxicity data were collected retrospectively, the number of evaluable patients varied across adverse events. P values were calculated using the Fisher–Freeman–Halton exact test with Monte Carlo simulation.

## Discussion

In this retrospective cohort study, we compared the outcomes of three adjuvant treatment approaches, defined according to the systemic chemotherapy regimen administered, and evaluated the prognostic significance of clinicopathological factors in patients with resected stage I–III gastric cancer. The main findings were as follows: (i) pathological stage and perineural invasion were independently associated with survival outcomes; (ii) no statistically significant differences in DFS or OS were observed between FUFA and XELOX in unadjusted pairwise analyses in the overall cohort; (iii) no significant differences in DFS or OS were observed between XELOX and FUFA in patients with node-positive disease; and (iv) patients treated with cisplatin-based chemotherapy had shorter survival in unadjusted analyses and higher rates of selected treatment-related toxicities.

Gastric cancer remains a disease with a substantial risk of recurrence even after curative resection, and the role of adjuvant treatment has been established by several randomized studies. The Intergroup 0116 trial demonstrated a survival benefit with postoperative chemoradiotherapy using fluorouracil and leucovorin compared with surgery alone and established postoperative chemoradiotherapy as a standard treatment option, particularly in patients who had not undergone an adequate lymphadenectomy (12, 13). The FUFA regimen used in our study was based on the postoperative chemoradiotherapy protocol used in the Intergroup 0116 trial. However, FUFA is not a contemporary standard adjuvant chemotherapy regimen, and its use in our cohort primarily reflects historical treatment practice during the earlier part of the study period rather than current guideline-based recommendations. Direct comparison of toxicity with the Intergroup 0116 trial is difficult because adverse-event data in our cohort were collected retrospectively and were incomplete.

Subsequent randomized trials have shown that adjuvant chemotherapy improves outcomes after curative gastrectomy. The ACTS-GC and JACCRO GC-07 trials demonstrated the efficacy of S-1-based treatment strategies, whereas the CLASSIC trial established XELOX as an effective adjuvant regimen after D2 gastrectomy (8, 10, 11, 14, 15). Based on these studies, XELOX and S-1-based regimens have become widely used adjuvant treatment options in Asian countries and are incorporated into international treatment guidelines (16–18). Stage I patients represented only 6.9% of the study population. Their inclusion reflects historical real-world postoperative treatment practice over the long study period rather than contemporary treatment recommendations. Because of the retrospective nature of the study, the specific rationale for administering adjuvant treatment to individual stage I patients could not be reliably reconstructed.

In the present study, no significant differences in DFS or OS were observed between the FUFA and XELOX groups in unadjusted pairwise analyses. Similarly, in the exploratory subgroup analysis of patients with node-positive disease, the differences between XELOX and FUFA did not reach statistical significance for either DFS or OS (p=0.088 and p=0.132, respectively). However, because of the retrospective design and the imbalances in baseline characteristics among treatment groups, these findings should be interpreted with caution.

Patients treated with cisplatin-based chemotherapy had shorter survival in unadjusted analyses and higher rates of neutropenia and diarrhea. Interpretation of these findings requires caution because most patients in this group received chemoradiotherapy and the baseline characteristics differed from those of the other treatment groups. Accordingly, the observed differences may reflect patient selection and treatment heterogeneity rather than differences in the efficacy of the regimens themselves. Therefore, the survival differences observed in this study should not be interpreted as evidence of the superiority or inferiority of any particular regimen.

Our findings regarding prognostic factors are largely consistent with previous studies. Advanced stage, lymphovascular invasion, perineural invasion, and extracapsular extension have all been associated with adverse outcomes in gastric cancer (7, 19–24). In the present study, pathological stage and perineural invasion remained independently associated with both DFS and OS, further supporting their prognostic significance. ECOG performance status ≥2 and R1 resection were also associated with shorter DFS and OS in univariable analyses, although neither variable was retained in the final multivariable models.

Several studies have suggested that a greater number of dissected lymph nodes is associated with improved survival (25–27). In contrast, we did not observe a significant association between the number of dissected lymph nodes and survival outcomes. This finding should be interpreted cautiously because detailed information on the extent of lymphadenectomy was not consistently available.

Similarly, previous studies have reported comparable survival outcomes after subtotal and total gastrectomy, although some studies have suggested poorer outcomes after total gastrectomy (28–32). In our cohort, patients who underwent subtotal gastrectomy had longer DFS and OS than those who underwent total gastrectomy. This association may reflect differences in tumor location and disease burden rather than the effect of the surgical procedure itself.

The present study has several limitations. First, its retrospective and single-center design may have introduced selection bias. Comorbidity burden was not systematically recorded and could not be included in the analyses, which may have contributed to residual confounding in treatment selection. Treatment discontinuation was associated with shorter survival in univariable analyses. However, treatment completion was not included in the multivariable models because some patients discontinued treatment after disease progression or died before completing treatment. Second, treatment groups were imbalanced with respect to several clinicopathological characteristics. Because of these baseline differences, residual confounding cannot be excluded, and the comparative efficacy analyses should be regarded as hypothesis-generating rather than definitive. Chemoradiotherapy was used in a high proportion of patients and at different rates across treatment groups, so the observed survival differences cannot be attributed to the systemic chemotherapy regimen alone. In addition, the small number of patients, particularly in the cisplatin-based group, did not allow a reliable propensity score–matched analysis. Third, the extent of lymphadenectomy (D1/D1+/D2) was not consistently documented and could not be reliably assessed. Treatment approaches changed over the long study period, and because regimen use was closely linked to treatment era, changes in surgical practice, staging, supportive care, and follow-up may have contributed to residual confounding. A sensitivity analysis in which treatment period was forced into the multivariable models did not attenuate the point estimate for XELOX compared with FUFA. Nevertheless, because treatment approach and treatment period were highly correlated, with almost all XELOX-treated patients treated after 2016, the independent effects of regimen choice and temporal changes in clinical practice could not be reliably disentangled. The treatment-period-adjusted estimates should therefore be interpreted cautiously. The within-period comparison was also inconclusive because only 11 patients received FUFA in 2017–2022, resulting in wide confidence intervals. These findings may not be generalizable to institutions with different treatment selection practices or to patients treated with current adjuvant regimens. The small size of the cisplatin-based group also limited the statistical power of comparisons involving this regimen. Follow-up durations also differed among treatment groups, which may have influenced survival estimates. Detailed information on chemotherapy dose reductions was not consistently available. Finally, some non-hematological toxicities may have been underreported because toxicity data were collected retrospectively.

Nevertheless, our findings add to the limited evidence comparing commonly used adjuvant treatment approaches (33–40) and provide additional information on prognostic factors in resected gastric cancer.

## Conclusions

In this retrospective cohort of patients with resected stage I–III gastric cancer, no statistically significant differences in DFS or OS were observed between FUFA and XELOX in unadjusted pairwise comparisons in the overall study population or in the exploratory analysis of patients with node-positive disease. However, in the primary multivariable models, XELOX was associated with lower hazards of recurrence and mortality than FUFA. When treatment period was forced into the models, the association with DFS was no longer statistically significant, whereas the association with OS remained significant. This discordance for DFS should be interpreted cautiously because treatment approach and treatment period were closely linked, making it difficult to separate the effect of regimen choice from temporal changes in clinical practice. Pathological stage and perineural invasion remained independently associated with survival outcomes. Although patients in the cisplatin-based treatment group had shorter survival in unadjusted analyses, no significant difference was observed between the cisplatin-based approach and FUFA after multivariable adjustment. The higher rates of neutropenia and diarrhea observed in the cisplatin-based group should be interpreted cautiously because toxicity data were collected retrospectively and were incomplete. These findings provide additional information on postoperative treatment strategies and should be validated in prospective multicenter studies.

## Data Availability

The raw data supporting the conclusions of this article will be made available by the authors, without undue reservation.
